# Regulation of CXCL1 chemokine and CSF3 cytokine levels in myometrial cells by the MAFF transcription factor

**DOI:** 10.1111/jcmm.14136

**Published:** 2019-01-22

**Authors:** James Saliba, Baptiste Coutaud, Vera Solovieva, Fangshi Lu, Volker Blank

**Affiliations:** ^1^ Lady Davis Institute for Medical Research Montreal Quebec Canada; ^2^ Department of Medicine McGill University Health Centre Montreal Quebec Canada; ^3^ Department of Physiology McGill University Montreal Quebec Canada

**Keywords:** CSF3, CXCL1, cytokines, IL1B, MAFF, myometrium

## Abstract

Cytokines play key roles in a variety of reproductive processes including normal parturition as well as preterm birth. Our previous data have shown that MAFF, a member of the MAF family of bZIP transcription factors, is rapidly induced by pro‐inflammatory cytokines in PHM1‐31 myometrial cells. We performed loss‐of‐function studies in PHM1‐31 cells to identify MAFF dependent genes. We showed that knockdown of MAFF significantly decreased CXCL1 chemokine and CSF3 cytokine transcript and protein levels. Using chromatin immunoprecipitation analyzes, we confirmed *CXCL1* and *CSF3* genes as direct MAFF targets. We also demonstrated that MAFF function in PHM1‐31 myometrial cells is able to control cytokine and matrix metalloproteinase gene expression in THP‐1 monocytic cells in a paracrine fashion. Our studies provide valuable insights into the MAFF dependent transcriptional network governing myometrial cell function. The data suggest a role of MAFF in parturition and/or infection‐induced preterm labour through modulation of inflammatory processes in the microenvironment.

## 1 INTRODUCTION

Due to lack of direct testing it is still not completely clear how human parturition is initiated. Current accepted theories for initiation of labour include a change in hormonal/paracrine regulation and the appearance of inflammation.^1^, ^2^ It has been suggested that the uterus at term constitutes an “inflammatory microenvironment” with leucocytes infiltrating the myometrium, uterine cervix and foetal membrane.^3^, ^4^, ^5^ Myometrial cells, in response to inflammation, secrete and stimulate surrounding cells to produce cytokines, including IL1B and IL6.^6^ Leucocytes, recruited by these cytokines, secrete both monocyte recruiting chemo attractants such as CCL2,^7^ as well as matrix metalloproteinase (MMPs), enzymes that are capable of digesting the extracellular matrix,^8^ thus contributing along with the cytokines to the activation of genes that ultimately promote cervical dilation/ripening, membrane rupture^9^ and contractions.^10^ Expression array studies established a network of genes modulated by IL1B in PHM1‐31 myometrial cells. The identified differentially expressed genes are associated with cell adhesion/cell motility, inflammation/immune response and proteolysis.^11^

Previous studies have linked the MAFF transcription factor to the inflammatory response.^11^, ^12^ MAFF belongs to the MAF (avian muscoloaponeurotic fibrosarcoma) basic leucine zipper (bZIP) family of transcription factors. The MAFs are classified into two subgroups. Large MAFs, comprising an activation domain at the N‐terminus, involved in the regulation of gene expression of various cell types and tissues including haematopoietic cells, bone, kidney, photoreceptor cells, as well as in oncogenesis.^13^, ^14^ The small MAFs, MAFF, MAFK^15^ and MAFG^16^ are lacking an obvious transactivation domain, but nevertheless playing key roles in transcriptional regulation.^17^, ^18^ Small MAF proteins can form homodimers through which they may function as transcriptional repressors by acting as dominant negative factors^19^ or heterodimerize with other bZIP proteins including the cap “n” collar (CNC) family members NFE2,^20^ NFE2L1,^21^ NFE2L2,^22^ NFE2L3,^23^, ^24^ BACH1 and BACH2.^25^ The resulting homo‐ or heterodimers bind to palindromic Maf recognition element (MARE) sequences, NF‐E2 binding motifs, antioxidant/electrophile response elements (ARE/EpRE) and CNC‐sMAF binding elements (CsMBE).^13^, ^17^, ^26^, ^27^ The small MAFs play important roles in the control of mammalian gene expression, and have been linked to a variety of pathways and/or pathologies,^17^, ^18^, ^28^ including cellular stress response and detoxification,^29^ diabetes,^30^ neuronal disease^31^ and cancer.^32^, ^33^

Previously, we found that *MAFF* transcript and protein levels are induced by the pro‐inflammatory cytokine IL1B and TNF alpha in PHM1‐31 myometrial cells.^11^, ^12^ MAFF was the only small MAF protein to be induced by cytokines, suggesting a specific role for this protein in the inflammatory response in uterine smooth muscle cells.^12^ An earlier report showed that *MAFF* transcripts are present in human term myometrium, but not in early gestation period or non‐pregnant myometrium.^34^

In the current study, we further explored the link between pro‐inflammatory cytokines and myometrial cell function in PHM1‐31 cells. We used knockdown approaches to dissect the cytokine‐dependent regulatory network in this cellular model. Our studies showed that the MAFF transcription factor functions as an essential regulator of chemokine and cytokine genes in myometrial cells. This is of interest, as pro‐inflammatory cytokine signalling mediates crucial functions in normal and premature birth, thus a better understanding of the underlying molecular mechanisms may help in the prevention of preterm labour.

## 2 MATERIALS AND METHODS

### 2.1 Cells and cell culture

PHM1‐31 myometrial cells were provided by Dr. Barbara Sanborn (Colorado State University) and were maintained at 37°C in high‐glucose DMEM media (11965‐092; Invitrogen, Thermo Fisher Scientific, Waltham, MA, USA) containing 0.1 mg/mL Geneticin (450‐130‐QL; WISENT Inc., QC, Canada), 10% foetal bovine serum, 2 mmol/L l‐glutamine and 2% antibiotic‐antimycotic solution containing 5000 U/mL penicillin and 5000 U/mL streptomycin as previously described.^12^ Cells were passaged using 0.05% trypsin‐EDTA (25300‐054; Invitrogen). For time course studies, PHM1‐31 cells, untreated (control) or treated with 10 ng/mL IL1B, were collected at different time points (0, 1, 3, 8 and 12 hours). PHM1‐31 cells were seeded at 6 × 10^4^/cm^2^ and scraped into PBS at 90% confluency for immunoblot analysis and collected by Trizol reagent (15596018; Invitrogen) for RNA extraction.

THP‐1 monocytic cells were provided by Dr. Andrew Mouland (Lady Davis Institute for Medical Research) and were maintained at 37°C in high‐glucose DMEM media (11965‐092; Invitrogen) containing 10% foetal bovine serum and 2% antibiotic‐antimycotic solution containing 5000 U/mL penicillin and 5000 U/mL streptomycin. The day of the experiment, THP‐1 cells were seeded at a density of 400 000 cells per well in a 12‐well tissue culture plate in 500 μL of media. Eight hours after change of media, 500 μL of supernatant derived from the culture of shRNA‐transduced PHM1‐31 cells were added for 24 hours to each well.

### 2.2 Lentivirus‐based transduction of cells with shRNA

Glycerol stocks of shRNA hairpins were obtained from the Sigma Mission library and isolation of plasmids was carried out with the PureLink^®^ HiPure Plasmid Maxiprep Kit (Invitrogen). HEK293T cells were seeded 24 hours before transfection. For each 10‐cm dish, 0.5 mL 2xHeBS (274 mmol/L NaCl, 10 mmol/L KCl, 1.5 mmol/L Na_2_HPO_4_·2H_2_O, 12 mmol/L dextrose, and 50 mmol/L Hepes in 500 mL MilliQ water at pH 7.01) was added into a sterile Eppendorf tube. In another sterile Eppendorf tube, 3 μg of plasmid DNA of interest, 2 μg of packaging vector pCMV dR8.91, 1 μg of VSV‐G envelope vector, 60 μL of 2 mol/L CaCl_2_ and distiled water were added to bring up the volume to 0.5 mL. The CaCl_2_/plasmid DNA mix was added to the 2xHeBS, incubated for 20 minutes and then added to the cells. Medium was refreshed after 16 hours. The supernatant of HEK293T cells containing lentivirus was collected after 24 hours to infect cells with 5 μg/mL polybrene (Millipore, Etobicoke, Canada) for 8 hours. The medium was refreshed after lentivirus infection and the cells were selected with puromycin. Individual shRNA vectors used were collected from the human TRC library (Sigma‐Aldrich Canada Cie., Oakville, Canada): TRC2 pLKO.5‐puro Non‐Target shRNA Control (NTC); shMAFF clone IDs TRCN0000415716 (sh1) and TRCN0000412857 (sh2).

### 2.3 Quantitative PCR

Total RNA was collected in Trizol reagent (15596018; Invitrogen) and extracted using Aurum™ Total RNA Mini Kit (Bio‐Rad Laboratories Ltd., Mississauga, Canada). cDNA was prepared using EasyScript Plus™ cDNA Synthesis Kit (Abmgood Applied Biological Materials Inc., Richmond, Canada) according to the manufacturer's instructions. Transcript abundance was determined by qPCR using SsoAdvanced SYBR Green supermix (Bio‐Rad) with the following primers purchased from Biorad: MAFF (qHsaCED0057106), CSF3 (qHsaCED0033948), CXCL1 (qHsaCED0046130), RPL37A (qHsaCED0005290) and IL6 (qHsaCID0020314). Primer sequences used for CCL2, MMP2 and MMP9 have been described.^35^ Additional custom primer sequences are listed in Table S1. The qPCR analysis was performed in a CFX96 Touch™ Real Time PCR detection system (Bio‐Rad). Data were analysed by the threshold cycle (Ct) comparative method. Samples were normalized to TBP and PPIA for PHM1‐31 extracts and to Beta Actin and RPL37A for THP‐1 extracts.^36^

### 2.4 Cell lysis and immunoblot analysis

Whole‐cell extracts were prepared by scraping cells using 1x PBS and cells were lysed for 10 minutes in whole‐cell lysis buffer (10 mmol/L Tris‐HCl pH 8.0, 420 mmol/L NaCl, 250 mmol/L sucrose, 2 mmol/L MgCl_2_, 1 mmol/L CaCl_2_, 1% Triton‐X100) supplemented with complete protease inhibitor cocktail (Roche, Mississauga, Canada, 04 693 116 001), and then centrifuged at 17 000 *g* for 10 minutes at 4°C. Supernatants were collected and protein concentrations were determined using a protein assay kit (Bio‐Rad, 500‐0006). Thirty microgram of the total protein lysate were separated by electrophoresis on a 15% sodium dodecyl sulphate polyacrylamide gel and transferred to a polyvinylidene difluoride membrane (Millipore). Blots were blocked using 5% milk in TBST (500 mmol/L Tris pH 7.6, 2 mol/L NaCl, 0.5% Tween) at room temperature for at least 1 hour and then incubated overnight at 4°C with primary antibodies specific for MAFF (1:1000)^12^ or ACTIN (1:50 000; Sigma A5441). Horseradish‐peroxidase (HRP)‐conjugated antibodies were used for 1 hour at room temperature. A goat antimouse secondary (1:30 000; Thermo Scientific, 31430) was used to detect ACTIN; a goat anti‐rabbit antibody (1:30 000; Thermo Scientific, 31460) was used to detect MAFF. The antigen‐antibody complexes were visualized using the chemiluminescent HRP substrate (Millipore, WBKLS0500) following the manufacturer's instructions and exposed to Hyperfilm (GE Healthcare, Baie‐d'Urfé, QC, Canada, 28‐9068‐35).

### 2.5 Chemokine and cytokine assays

ELISA assays were carried out using human CXCL1/GRO alpha (R&D Systems, DY275‐05) and G‐CSF (CSF3) DuoSet (R&D Systems, Inc., Minneapolis, MN, USA, DY214‐05) ELISAs according to the manufacturer's instructions. Plates were prepared by coating the capture antibody (mouse anti‐human GROα at 4 μg/mL and human G‐CSF at 1 μg/mL) overnight at room temperature followed by three washes with PBS. Subsequently, plates were blocked for at least an hour with reagent diluent followed by another three washes. For the assay procedure, detection antibodies were used at 40 ng/mL for biotinylated goat anti‐human GROα, at 300 ng/mL for the human G‐CSF, and streptavidin‐HRP was used at a 40‐fold dilution into reagent diluent. The optical density of each well was calculated using a microplate reader (Fluostar Optima; BMG Labtech, Cary, NC, USA) by subtracting the absorbance reading measured at 600 nm (background) from the one obtained at 450 nm.

### 2.6 Chromatin immunoprecipitation

ChIP assays were carried out using SimpleChIP^®^ Enzymatic Chromatin IP Kit (Cell Signaling Technology, Inc., Danvers, MA, USA, 9003) according to the manufacturer's instructions. 3 × 10^6^ PHM1‐31 cells were used per IP. Briefly, cells were cross‐linked using 1% formaldehyde for 10 minutes at room temperature and quenched with glycine to a final concentration of 0.125 mol/L for 5 minutes. Subsequently, DNA was lysed for 20 minutes at 37°C using 0.2 μL of micrococcal nuclease (Cell Signaling, 10011). Digestion was stopped by adding 10 μL of 0.5 mol/L EDTA (Cell Signaling, 7011) per IP prep on ice for 2 minutes. This was followed by nuclei lysis with three sets of 20‐second pulses at 15% intensity using a Fisher Scientific model 500 sonic dismembrator. The samples were then incubated overnight with ChIP‐Grade Protein G Magnetic Beads (Cell Signaling, 9006) with an antibody specific for MAFF.^12^ Flag rabbit IgG (DYKDDDDK Tag antibody; Cell Signaling, 2368S) was used as a negative control. After stringent washes, chromatin was eluted in ChIP Elution Buffer (Cell Signaling, 7009). Eluates were reverse cross‐linked by adding 6 μL 5 mol/L NaCl and 2 μL Proteinase K (Cell Signaling, 10012), and incubated for 2 hours at 65°C. DNA was extracted and purified using Spin Columns (Cell Signaling, 10010) and eluted in DNA Elution Buffer (Cell Signaling, 10009). ChIP‐qPCR analyses were performed in a CFX96 Touch™ Real Time PCR detection system (Bio‐Rad) and the primers used are listed in Table S1.

### 2.7 Statistical analysis

Data are presented as mean ± standard error of the mean (SEM). Differences between experiments were calculated with two‐tailed Student *t*‐test, for at least three independent experiments and statistically significant data are indicated. One asterisk indicates *P* < 0.05, two asterisks indicate *P* < 0.01 and three asterisks indicate *P* < 0.001.

## 3 RESULTS

### 3.1 CXCL1 and CSF3 levels are controlled by MAFF

**Figure 1:**
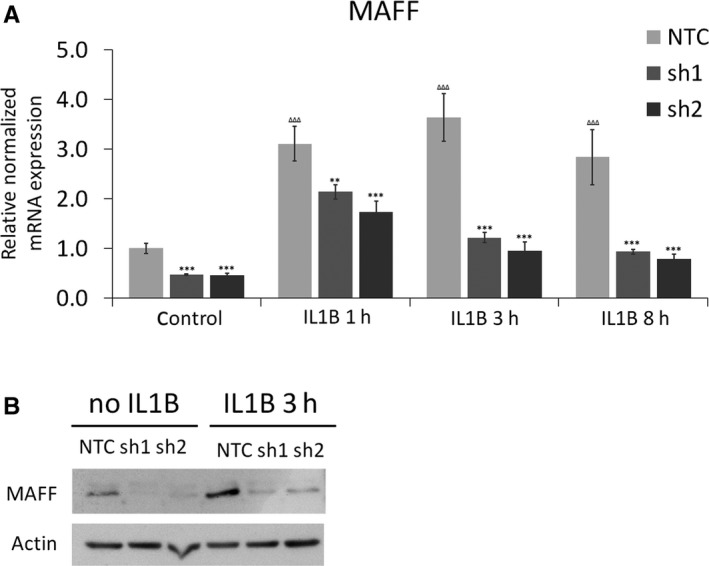
Confirmation of MAFF knockdown in PHM1‐31 myometrial cells. PHM1‐31cells were transduced with shRNA (NTC, sh1 or sh2) and MAFF knockdown analysed in the presence or absence of IL1B (10 ng/mL). (A) MAFF knockdown at the transcript level was quantified by quantitative PCR after no‐, 1, 3 or 8 h of IL1B treatment. (B) MAFF knockdown at the protein level was detected by immunoblot by using antibodies against MAFF or actin to control for protein loading after no‐ or 3 h of IL1B treatment. Data represent mean values ± SEM; unpaired *t*‐test; *P < 0.05; **P < 0.01; ***P < 0.001, ΔΔΔ *P* < 0.001. Asteriks correspond to the significance with the control (NTC) at the same time point and triangles to the significance between controls

**Figure 2:**
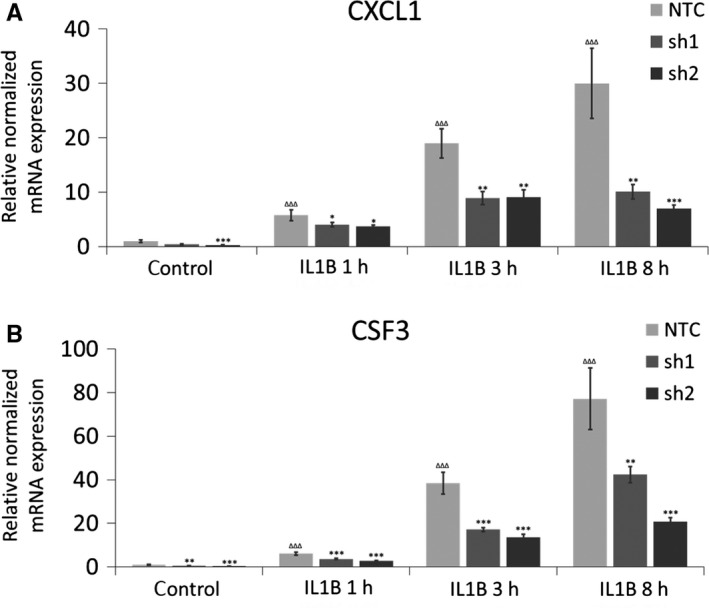
Control of CXCL1 and CSF3 transcript levels by MAFF in PHM1‐31 myometrial cells. PHM1‐31cells were transduced with shRNA (NTC, sh1 or sh2) and treated (or not) with 10 ng/mL IL1B for 1, 3 or 8 h. The regulation of CXCL1 (A) and CSF3 (B) mRNA expression upon MAFF knockdown was quantified by quantitative PCR. Data represent mean values ± SEM; unpaired *t*‐test; *P < 0.05; **P < 0.01; ***P < 0.001, ΔΔΔ *P* < 0.001. Asterisks correspond to the significance with the control (NTC) at the same time point and triangles to the significance between controls

MAFF transcripts are highly expressed in term myometrium, but not during early gestation periods and in non‐pregnant myometrium.^34^ Our previous data showed that pro‐inflammatory cytokines including IL1B rapidly induce MAFF transcript and protein levels in human PHM1‐31 myometrial cells,^12^ and that a larger network of inflammatory genes is equally modulated by IL1B.^11^ We thus hypothesized that the MAFF transcription factor is involved in the regulation of uterine smooth muscle genes linked to inflammation at the onset of parturition. We chose to pursue our studies in PHM1‐31 cells, a myometrial cell model we have analysed previously^11^, ^12^ and for which we optimized lentiviral based shRNA knockdown strategies for loss‐of‐function studies. We confirmed efficient shRNA mediated MAFF knockdown at the transcript and protein level in these cells with or without IL1B treatment (Figure 1A,B). We also observed a prominent increase of MAFF mRNA and protein upon IL1B treatment, confirming our previous data. With respect to MAFF targets, we focused on genes coding for CXCL1 chemokine and CSF3 cytokine, as our earlier results had shown that they are also significantly induced by IL1B^11^ and since they have been shown to be either directly or indirectly involved in the processes leading to parturition.^37^, ^38^, ^39^, ^40^, ^41^ Indeed, the knockdown of MAFF resulted in a significant decrease of *CXCL1* and *CSF3* gene transcripts under basal conditions and following IL1B treatment for 1, 3 and 8 hours (Figure 2A,B), suggesting that MAFF is involved in the control of the expression of these genes. In the non‐targeting control samples, both, *CXCL1* and *CSF3* mRNA levels are strongly increased upon IL1B treatment in a time dependent manner, emphasizing the role of MAFF in the regulation of these genes upon IL1B induction (Figure 2A).

**Figure 3:**
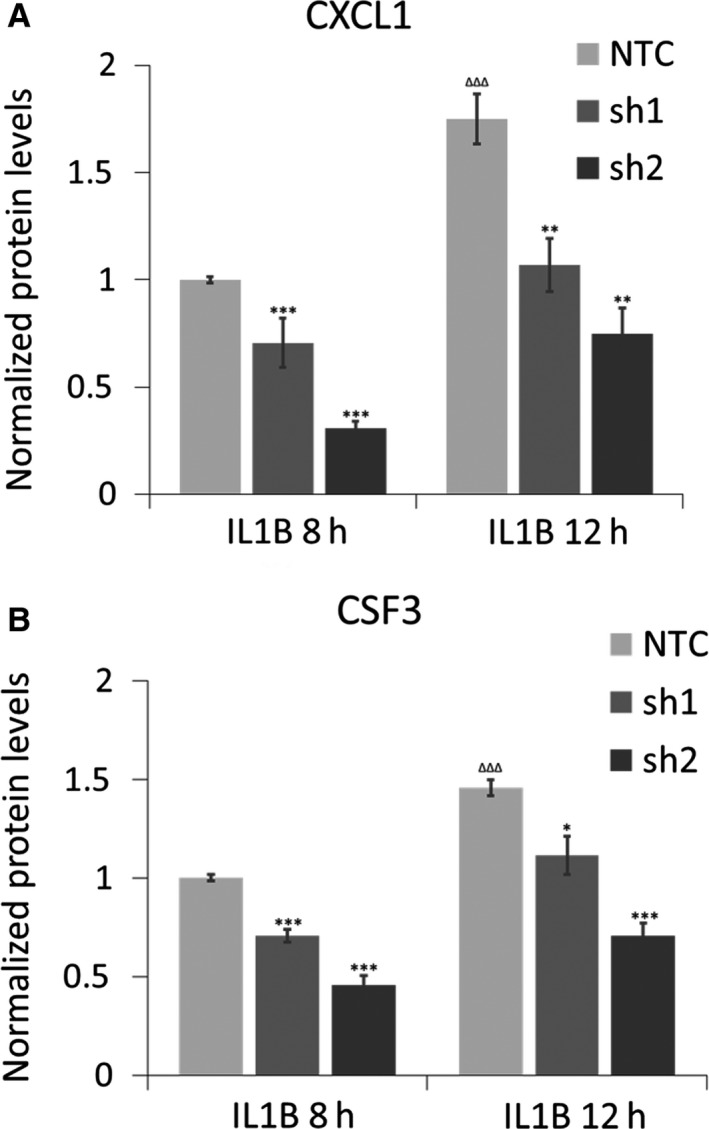
Regulation of CXCL1 and CSF3 protein levels by MAFF in PHM1‐31 myometrial cells. PHM1‐31cells were transduced with shRNA (NTC, sh1 or sh2) and treated with 10 ng/mL IL1B for 8 or 12 h. The regulation of CXCL1 chemokine secretion (A) and CSF3 cytokine secretion (B) upon MAFF knockdown was quantified by ELISA assays. Data represent mean values ± SEM; unpaired *t*‐test; *P < 0.05; **P < 0.01; ***P < 0.001, ΔΔΔ *P* < 0.001. Asterisks correspond to the significance with the control (NTC) at the same time point and triangles to the significance between controls

In additional studies, we performed ELISA assays to confirm our data at the protein level. CXCL1 chemokine and CSF3 cytokine secretion are greatly reduced upon MAFF knockdown in PHM1‐31 cells following 8 and 12 hours of IL1B treatment (Figure 3A,B). Protein levels without IL1B treatment were below the sensitivity threshold of the ELISA kits used. Taken together, these data suggest that MAFF positively controls CXCL1 and CSF3 expression in PHM1‐31 myometrial cells both in the presence and absence of IL1B.

### 3.2 Binding of MAFF transcription factor to the *CXCL1* and *CSF3* loci

**Figure 4:**
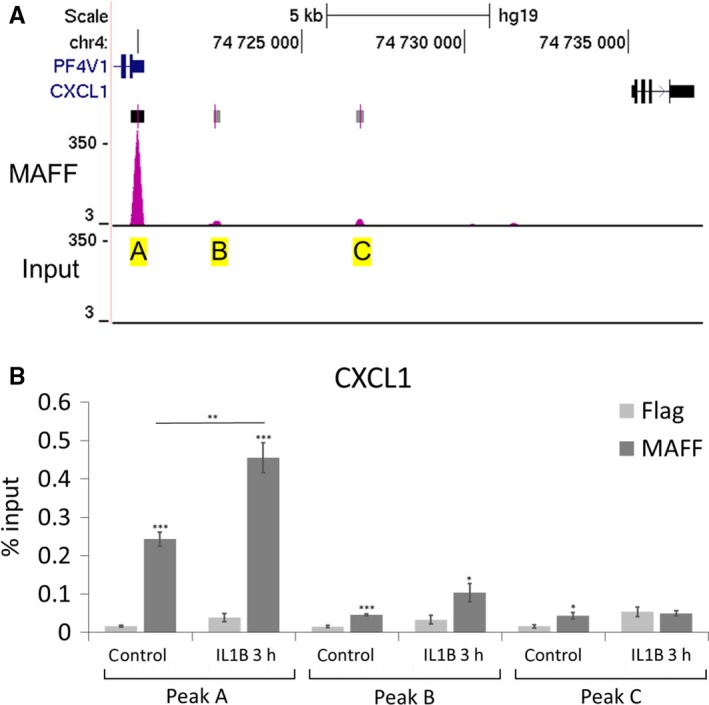
Binding of MAFF transcription factor to CXCL1 loci in PHM1‐31 myometrial cells. (A) Location of MAFF binding sites at the CXCL1 loci were established using the UCSC genome browser database and data from the ENCODE consortium (PMID: 22955616), SYDH track (GEO:GSM935306) showing MAFF specific binding to CXCL1 loci in HEPG2 cells. Chosen peaks for CXCL1 (A‐C) are marked in yellow, rectangles on top of peaks represent statistically significant peak calls, the darker rectangles being more significant. (B) ChIP analysis of MAFF transcription factor binding to CXCL1 loci. PHM1‐31 cells were treated (or not) with 10 ng/mL IL1B for 3 h and analysed by chromatin immunoprecipitation using antibodies against MAFF or FLAG to control for non‐specific binding. Data represent mean values ± SEM; unpaired *t*‐test; *P < 0.05; **P < 0.01; ***P < 0.001

**Figure 5:**
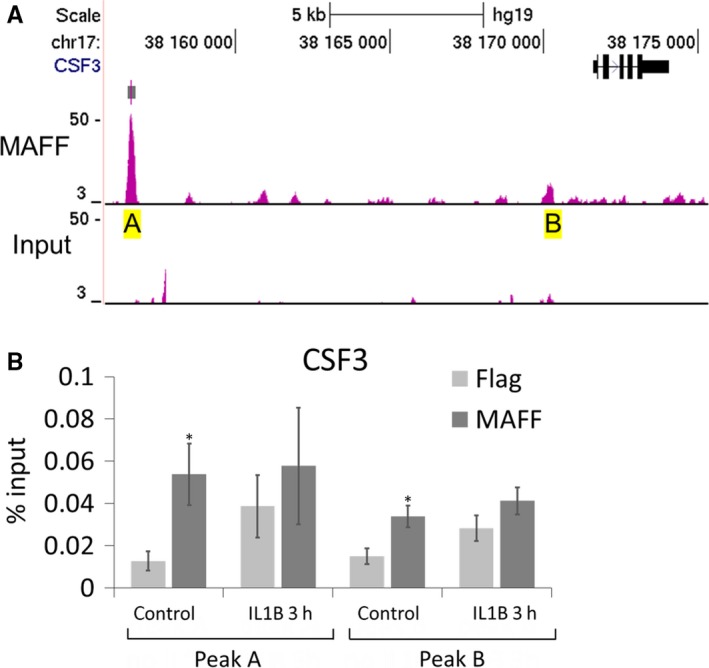
Binding of MAFF transcription factor to CSF3 loci in PHM1‐31 myometrial cells. (A) Location of MAFF binding sites at the CSF3 loci were established using the UCSC genome browser database and data from the ENCODE consortium (PMID: 22955616), SYDH track (GEO:GSM935306) showing MAFF specific binding to CSF3 loci in HEPG2 cells. Chosen peaks for CSF3 (A and B) are marked in yellow. (B) ChIP analysis of MAFF transcription factor binding to CSF3 loci. PHM1‐31 cells were treated (or not) with 10 ng/mL IL1B for 3 h and analysed by chromatin immunoprecipitation using antibodies against MAFF or FLAG to control for non‐specific binding. Data represent mean values ± SEM; unpaired *t*‐test; *P < 0.05; **P < 0.01; ***P < 0.001

To determine whether MAFF recognizes specific sequences in the *CXCL1* and *CSF3* genes, we searched for specific DNA binding peaks in UCSC genome browser database.^42^ Data from the ENCODE consortium,^42^ SYDH track (GEO: GSM935306), showed MAFF specific binding to *CXCL1* and *CSF3* loci in HEPG2 hepatocellular carcinoma cells. We examined three DNA regions of interest (A, B and C) for *CXCL1* corresponding to the three peaks showing significant MAFF binding in the *CXCL1* loci in this cell line (Figure 4A). For CSF3, two DNA regions were chosen, A and B. While peak A is the most significant binding peak in HEPG2 cells, peak B is the second strongest and located in the proximity of the *CSF3* loci (Figure 5A). We performed ChIP assays examining those regions and found that MAFF binds to the analysed genomic regions in both the *CXCL1* and *CSF3* genes in a significant manner in PHM1‐31 cells (Figures 4B and 5B). For the *CXCL1* locus, we observed significant binding of MAFF to all three selected regions without IL1B treatment. Upon induction with IL1B, each peak displayed a different result. Peak A shows a significant increase of MAFF binding which establishes a direct transcriptional effect of MAFF on CXCL1 regulation. Peak B displayed a trend while peak C lost significance for MAFF binding. For the *CSF3* gene, we noted significant binding of MAFF in both of the identified regions without IL1B treatment and a trend that fails to reach statistical significance when treated with IL1B. Contrary to CXCL1, there is no change in MAFF binding upon IL1B treatment for CSF3 suggesting a direct regulation but through an IL1B independent mechanism. Our data suggest that *CXCL1* chemokine and *CSF3* cytokine loci are direct targets of the MAFF transcription factor.

### 3.3 Effect of MAFF activity in myometrial cells on monocyte gene expression

**Figure 6:**
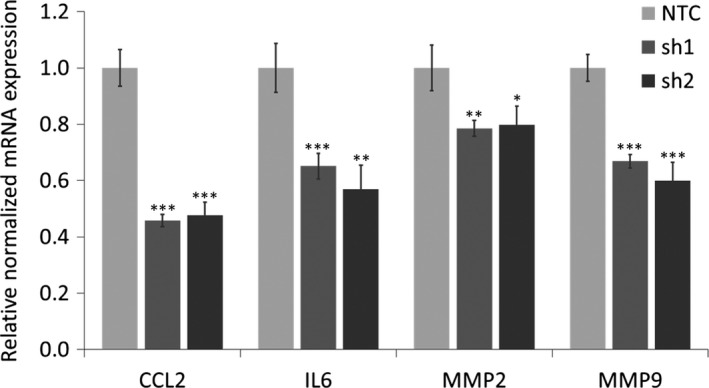
Effect of MAFF mediated regulation in PHM1‐31 cells on THP‐1 monocytes. THP 1 cells were cultured for 24 h with supernatant from PHM1‐31 cells transduced with NTC, sh1 or sh2. The regulation of CCL2, IL6, MMP2 and MMP9 mRNA expression was quantified by quantitative PCR. Data represent mean values ± SEM; unpaired *t*‐test; *P < 0.05; **P < 0.01; ***P < 0.001

It has been proposed that labour resembles an inflammatory process, with the activation of leucocytes from maternal tissue as well as the increasing levels of cytokines and chemokines from gestational tissue.^43^ Thus, we examined whether regulation of MAFF expression in PHM1‐31 cells might have an impact on the leucocyte environment. As a study model, we chose human THP‐1 monocytic cells.^44^ We investigated the expression of *CCL2* and *IL6* cytokine genes that are highly up‐regulated in vivo in labouring myometrium as well as the genes coding for two monocyte activation markers, *MMP2* and *MMP9* that contribute to cervical ripening.^35^ We treated THP‐1 cells for 24 hours with supernatant from PHM1‐31 cells that have been transduced with control or MAFF shRNAs. MAFF knockdown leads to a significant decrease in CCL2, IL6, MMP2 and MMP9 transcript levels (Figure 6A‐D). Our results indicate that MAFF transcription factor activity in myometrial cells is able to control cytokine and activation marker gene expression levels in neighbouring monocytes in a paracrine fashion.

## 4 DISCUSSION

Our previous data linked the MAFF transcription factor to pro‐inflammatory cytokine signaling in uterine smooth muscle cells.^11^, ^12^ It has been established that at parturition, leucocytes invade the uterus and together with the local cells secrete cytokines, thus creating an inflammatory microenvironment in the reproductive tissues.^3^ This process is essential for membrane rupture, cervical dilation, myometrial contraction and expulsion of the foetus.^1^ In earlier studies, it has been reported that MAFF is present at high levels in pregnant myometrium at term.^34^ Using PHM1‐31 uterine smooth muscle cells as well as primary myometrial cells as models, we previously showed that MAFF transcript and protein levels are up‐regulated by IL1B and TNF,^11^, ^12^ both of which have been linked to the onset of labour.^45^ The MAFF gene is highly sensitive to IL1B and the induction is very rapid, as MAFF mRNA increases are observed at as low as 0.05 ng/mL of the cytokine and as early as 30 minutes following the treatment.^12^ We also found that MAFF is the only member of the small MAF family, to be regulated by IL1B and TNF in this cell type.^12^ Together, these studies suggested a specific role for MAFF in controlling inflammatory processes in myometrial cells. In the present study, we identified the genes coding for CXCL1 chemokine and CSF3 cytokine as MAFF targets use a loss‐of‐function approach. Transduction of lentiviral vectors coding for MAFF specific shRNAs resulted in a substantial decrease of MAFF expression, both in the absence and the presence of IL1B. MAFF knockdown led to a significant down‐regulation of CXCL1 and CSF3, both at the transcript and protein levels, suggesting that these genes are positively controlled by the transcription factor. Of interest, in a previous study using genechip expression array profiling in PHM1‐31 cells, we had identified CXCL1 and CSF3 as one of the transcripts that were most strongly induced by IL1B.^11^ Here, we confirmed that IL1B induces both genes. In the context of preterm labour, induction of pro‐inflammatory cytokine levels by infection or stress may thus precede the activation of MAFF. A series of studies have identified IL1B as one of the cytokines playing a key role in the induction of normal parturition as well as preterm labour.^4^, ^46^, ^47^, ^48^ Importantly, CXCL1 and CSF3 have been found to be up‐regulated during spontaneous labour as demonstrated in the RNA‐seq transcriptome studies comparing patient samples prior to and after the onset of spontaneous labour,^49^ clearly linking these proteins to the labour process. In addition, CXCL1 and CSF3 have been shown to be important for neutrophil activation and recruitment,^50^, ^51^, ^52^ thus the control of myometrial gene expression by MAFF may play a role in normal parturition and/or preterm labour by promoting a pro‐inflammatory microenvironment.

One important question was whether MAFF, as a transcription factor, directly controls the expression of the identified targets. We consulted UCSC browser data of genome wide MAFF binding analyses in HEPG2 cells and identified a series of significant MAFF DNA binding peaks present in the CXCL1 and CSF3 loci. Our data showed that MAFF is binding to these regions in PHM1‐31 cells, and that the binding preference varies depending on the peak region analysed. As we based our analysis on ChIP‐sequencing data generated in hepatic cells, it is conceivable that in our myometrial cell model MAFF may also bind to other sequences in these genes. We conclude that MAFF acts as a direct upstream regulator of the transcription of these genes.

As CXCL1 and CSF3 are both secreted proteins, we hypothesized that regulation of myometrial gene expression by MAFF may contribute to inflammatory processes in the microenvironment. Indeed, we found that MAFF knockdown in PHM1‐31 cells can also affect the gene expression pattern in THP‐1 monocyte cells in a paracrine manner. MAFF levels in myometrial cells are able to mediate the regulation of CCL2 chemokine, IL6 cytokine and activation makers such as MMP2 and MMP9 in monocytes. This is of interest, as leucocyte infiltration of the uterine cervix, predominantly consisting of neutrophils and macrophages, coincides with the onset of labour.^53^ Others have observed phenotypic and metabolic changes in maternal neutrophils and granulocytes in preterm labour.^54^ Of note, CXCL1 has been shown to be involved in monocyte recruitment.^55^ Also, CSF3 functions as an important maturation factor for monocytes as well as granulocytes.^56^, ^57^, ^58^ In conclusion, our data further support the involvement of CXCL1 and CSF3 in monocyte and granulocyte recruitment and maturation, and establishes MAFF as a key upstream regulator of these cytokine‐mediated signaling pathways in myometrial cells, including the recruitment of leuuocytes, a critical step for the labour process.

**Figure 7:**
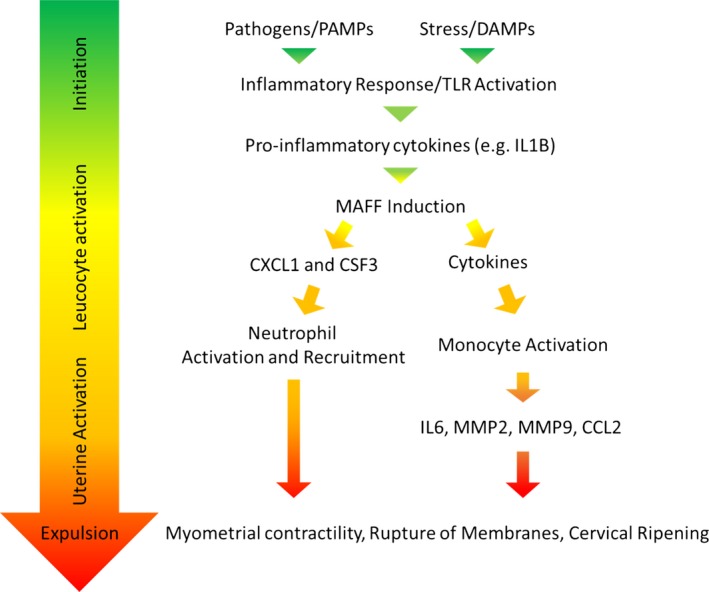
Model of chemokine and cytokine regulation by MAFF in parturition and labor

Based on our data, we propose a model (Figure 7), in which the MAFF transcription factor functions as a key regulator in the labour process, acting as a link between initiation of the process and the following activation of leucocytes and subsequent uterine activation.^40^, ^48^, ^59^, ^60^, ^61^ In future studies, it will be important to further dissect the MAFF‐mediated regulatory network in myometrial cells, including signaling pathways and interacting proteins that control the activity of the MAFF transcription factor as well as coordinated regulation of the labour process together with other transcriptional regulators. A better understanding of the MAFF regulatory network in parturition may provide means to prevent preterm labour.

## ACKNOWLEDGEMENTS

We wish to thank Joo Yeoun (Sophie) Park, Audrey Burban and Jennie Jackson for critical reading of the manuscript and/or fruitful discussions. We also would like to thank Barbara Sanborn for providing PHM1‐31 myometrial cells and Andrew Mouland for providing THP‐1 cells, and Anna Derjuga for expert technical assistance. We thank François Lefebvre for his help with the bioinformatics analysis of RNA sequencing data at an earlier stage. We would like to acknowledge the ENCODE project consortium and the Michael Snyder laboratory, Stanford for the MAFF ChIP‐seq data. This work was supported by SEG (Liban) and McGill Integrated Cancer Research Training Program (MICRTP) studentships to James Saliba, and a MICRTP studentship to Fangshi Lu. This research was funded by National Science Engineering Research Council of Canada (NSERC) RGPIN 238919‐08 and RGPIN 2017‐05735 grants to VB.

## CONFLICT OF INTEREST

The authors declare no conflict of interest.

## References

[1] Kamel RM . The onset of human parturition. Arch Gynecol Obstet. 2010;281:975‐982. 20127346 10.1007/s00404-010-1365-9

[2] Sivarajasingam SP , Imami N , Johnson MR . Myometrial cytokines and their role in the onset of labour. J Endocrinol. 2016;231:R101‐R119. 27647860 10.1530/JOE-16-0157

[3] Gomez‐Lopez N , Laresgoiti‐Servitje E , Olson DM , Estrada‐Gutierrez G , Vadillo‐Ortega F . The role of chemokines in term and premature rupture of the fetal membranes: a review. Biol Reprod. 2010;82:809‐814. 20089887 10.1095/biolreprod.109.080432

[4] Blank V , Hirsch E , Challis JRG , Romero R , Lye SJ . Cytokine signaling, inflammation and preterm labour: a workshop report. Placenta. 2008;29:S102‐S104. 18082255 10.1016/j.placenta.2007.10.011

[5] Romero R , Espinoza J , Goncalves LF , Kusanovic JP , Friel L , Hassan S . The role of inflammation and infection in preterm birth. Semin Reprod Med. 2007;25:21‐39. 17205421 10.1055/s-2006-956773 PMC8324073

[6] Li H , Yu Y , Shi Y , et al. HoxA13 stimulates myometrial cells to secrete IL‐1beta and enhance the expression of contraction‐associated proteins. Endocrinology. 2016;157:2129‐2139. 26982635 10.1210/en.2015-2005

[7] Shynlova O , Tsui P , Dorogin A , Lye SJ . Monocyte chemoattractant protein‐1 (CCL‐2) integrates mechanical and endocrine signals that mediate term and preterm labor. J Immunol. 2008;181:1470‐1479. 18606702 10.4049/jimmunol.181.2.1470

[8] Vadillo‐Ortega F , Estrada‐Gutierrez G . Role of matrix metalloproteinases in preterm labour. BJOG. 2005;112(Suppl 1):19‐22. 15715589 10.1111/j.1471-0528.2005.00579.x

[9] Cockle JV , Gopichandran N , Walker JJ , Levene MI , Orsi NM . Matrix metalloproteinases and their tissue inhibitors in preterm perinatal complications. Reprod Sci. 2007;14:629‐645. 18000225 10.1177/1933719107304563

[10] Voltolini C , Battersby S , Novembri R , et al. Urocortin 2 role in placental and myometrial inflammatory mechanisms at parturition. Endocrinology. 2015;156:670‐679. 25426872 10.1210/en.2014-1432

[11] Chevillard G , Derjuga A , Devost D , Zingg HH , Blank V . Identification of interleukin‐1beta regulated genes in uterine smooth muscle cells. Reproduction. 2007;143:811‐822. 10.1530/REP-07-0289 18042638

[12] Massrieh W , Derjuga A , Doualla‐Bell F , Ku CY , Sanborn BM , Blank V . Regulation of the MAFF transcription factor by proinflammatory cytokines in myometrial cells. Biol Reprod. 2006;74:699‐705. 16371591 10.1095/biolreprod.105.045450

[13] Blank V , Andrews NC . The Maf transcription factors: regulators of differentiation. Trends Biochem Sci. 1997;22:437‐441. 9397686 10.1016/s0968-0004(97)01105-5

[14] Eychene A , Rocques N , Pouponnot C . A new MAFia in cancer. Nat Rev Cancer. 2008;8:683‐693. 19143053 10.1038/nrc2460

[15] Fujiwara KT , Kataoka K , Nishizawa M . Two new members of the maf oncogene family, mafK and mafF, encode nuclear b‐Zip proteins lacking putative trans‐activator domain. Oncogene. 1993;8:2371‐2380. 8361754

[16] Kataoka K , Igarashi K , Itoh K , et al. Small Maf proteins heterodimerize with Fos and may act as competitive repressors of the NF‐E2 transcription factor [published erratum appears in Mol Cell Biol 1995 Jun; 15(6):3461]. Mol Cell Biol. 1995;15:2180‐2190. 7891713 10.1128/mcb.15.4.2180 PMC230446

[17] Blank V . Small Maf proteins in mammalian gene control: mere dimerization partners or dynamic transcriptional regulators? J Mol Biol. 2008;376:913‐925. 18201722 10.1016/j.jmb.2007.11.074

[18] Kannan MB , Solovieva V , Blank V . The small MAF transcription factors MAFF, MAFG and MAFK: current knowledge and perspectives. Biochim et Biophys acta ‐ Mol Cell Res. 2012;1823:1841‐1846. 10.1016/j.bbamcr.2012.06.012 22721719

[19] Igarashi K , Kataoka K , Itoh K , Hayashi N , Nishizawa M , Yamamoto M . Regulation of transcription by dimerization of erythroid factor NF‐E2 p45 with small Maf proteins [see comments]. Nature. 1994;367:568‐572. 8107826 10.1038/367568a0

[20] Andrews NC , Kotkow KJ , Ney PA , Erdjument‐Bromage H , Tempst P , Orkin SH . The ubiquitous subunit of erythroid transcription factor NF‐E2 is a small basic‐leucine zipper protein related to the v‐maf oncogene. Proc Natl Acad Sci U S A. 1993;90:11488‐11492. 8265578 10.1073/pnas.90.24.11488 PMC48009

[21] Johnsen O , Skammelsrud N , Luna L , Nishizawa M , Prydz H , Kolsto AB . Small Maf proteins interact with the human transcription factor TCF11/Nrf1/LCR‐F1. Nucleic Acids Res. 1996;24:4289‐4297. 8932385 10.1093/nar/24.21.4289 PMC146217

[22] Toki T , Itoh J , Kitazawa J , et al. Human small Maf proteins form heterodimers with CNC family transcription factors and recognize the NF‐E2 motif. Oncogene. 1997;14:1901‐1910. 9150357 10.1038/sj.onc.1201024

[23] Kobayashi A , Ito E , Toki T , et al. Molecular cloning and functional characterization of a new Cap'n’ collar family transcription factor Nrf3. J Biol Chem. 1999;274:6443‐6452. 10037736 10.1074/jbc.274.10.6443

[24] Chenais B , Derjuga A , Massrieh W , et al. Functional and placental expression analysis of the human NRF3 transcription factor. Mol Endocrinol. 2005;19:125‐137. 15388789 10.1210/me.2003-0379

[25] Oyake T , Itoh K , Motohashi H , et al. Bach proteins belong to a novel family of BTB‐basic leucine zipper transcription factors that interact with MafK and regulate transcription through the NF‐E2 site. Mol Cell Biol. 1996;16:6083‐6095. 8887638 10.1128/mcb.16.11.6083 PMC231611

[26] Motohashi H , Shavit JA , Igarashi K , Yamamoto M , Engel JD . The world according to Maf. Nucleic Acids Res. 1997;25:2953‐2959. 9224592 10.1093/nar/25.15.2953 PMC146842

[27] Otsuki A , Suzuki M , Katsuoka F , et al. Unique cistrome defined as CsMBE is strictly required for Nrf2‐sMaf heterodimer function in cytoprotection. Free Radic Biol Med. 2016;91:45‐57. 26677805 10.1016/j.freeradbiomed.2015.12.005

[28] Motohashi H , O'Connor T , Katsuoka F , Engel J , Yamamoto M . Integration and diversity of the regulatory network composed of Maf and CNC families of transcription factors. Gene. 2002;294:1‐12. 12234662 10.1016/s0378-1119(02)00788-6

[29] Yamazaki H , Katsuoka F , Motohashi H , Engel JD , Yamamoto M . Embryonic lethality and fetal liver apoptosis in mice lacking all three small Maf proteins. Mol Cell Biol 2011;32:808‐816. 22158967 10.1128/MCB.06543-11 PMC3272985

[30] van Oostrom O , de Kleijn DP , Fledderus JO , et al. Folic acid supplementation normalizes the endothelial progenitor cell transcriptome of patients with type 1 diabetes: a case‐control pilot study. Cardiovasc Diabetol. 2009;8:47. 19706161 10.1186/1475-2840-8-47 PMC2739843

[31] Katsuoka F , Motohashi H , Tamagawa Y , et al. Small Maf compound mutants display central nervous system neuronal degeneration, aberrant transcription, and Bach protein mislocalization coincident with myoclonus and abnormal startle response. Mol Cell Biol. 2003;23:1163‐1174. 12556477 10.1128/MCB.23.4.1163-1174.2003 PMC141134

[32] Amit I , Citri A , Shay T , et al. A module of negative feedback regulators defines growth factor signaling. Nat Genet. 2007;39:503‐512. 17322878 10.1038/ng1987

[33] Fang M , Ou J , Hutchinson L , Green MR . The BRAF oncoprotein functions through the transcriptional repressor MAFG to mediate the CpG Island Methylator phenotype. Mol Cell. 2014;55:904‐915. 25219500 10.1016/j.molcel.2014.08.010 PMC4170521

[34] Kimura T , Ivell R , Rust W , et al. Molecular cloning of a human MafF homologue, which specifically binds to the oxytocin receptor gene in term myometrium. Biochem Biophys Res Commun. 1999;264:86‐92. 10527846 10.1006/bbrc.1999.1487

[35] Srikhajon K , Shynlova O , Preechapornprasert A , Chanrachakul B , Lye S . A new role for monocytes in modulating myometrial inflammation during human labor. Biol Reprod. 2014;91:10. 24829032 10.1095/biolreprod.113.114975

[36] Maess MB , Sendelbach S , Lorkowski S . Selection of reliable reference genes during THP‐1 monocyte differentiation into macrophages. BMC Mol Biol. 2010;11:90. 21122122 10.1186/1471-2199-11-90 PMC3002353

[37] Gomez‐Lopez N , Tanaka S , Zaeem Z , Metz GA , Olson DM . Maternal circulating leukocytes display early chemotactic responsiveness during late gestation. BMC Pregnancy Childbirth. 2013;13(Suppl 1):S8. 23445935 10.1186/1471-2393-13-S1-S8 PMC3561147

[38] Hamilton SA , Tower CL , Jones RL . Identification of chemokines associated with the recruitment of decidual leukocytes in human labour: potential novel targets for preterm labour. PLoS ONE. 2013;8:e56946. 23451115 10.1371/journal.pone.0056946 PMC3579936

[39] Yellowhair TR , Noor S , Maxwell JR , et al. Preclinical chorioamnionitis dysregulates CXCL1/CXCR2 signaling throughout the placental‐fetal‐brain axis. Exp Neurol. 2018;301:110‐119. 29117499 10.1016/j.expneurol.2017.11.002

[40] Shynlova O , Nedd‐Roderique T , Li Y , Dorogin A , Lye SJ . Myometrial immune cells contribute to term parturition, preterm labour and post‐partum involution in mice. J Cell Mol Med. 2013;17:90‐102. 23205502 10.1111/j.1582-4934.2012.01650.x PMC3823139

[41] Pabona JM , Zhang D , Ginsburg DS , Simmen FA , Simmen RC . Prolonged pregnancy in women is associated with attenuated myometrial expression of progesterone receptor co‐regulator Kruppel‐like Factor 9. J Clin Endocrinol Metab. 2015;100:166‐174. 25313913 10.1210/jc.2014-2846 PMC4283014

[42] Consortium EP . An integrated encyclopedia of DNA elements in the human genome. Nature. 2012;489:57‐74. 22955616 10.1038/nature11247 PMC3439153

[43] Shynlova O , Lee YH , Srikhajon K , Lye SJ . Physiologic uterine inflammation and labor onset: integration of endocrine and mechanical signals. Rep Sci. 2013;20:154‐167. 10.1177/1933719112446084 22614625

[44] Lee YH , Shynlova O , Lye SJ . Stretch‐induced human myometrial cytokines enhance immune cell recruitment via endothelial activation. Cell Mol Immunol. 2015;12:231‐242. 24882387 10.1038/cmi.2014.39 PMC4654292

[45] Cookson VJ , Chapman NR . NF‐kappaB function in the human myometrium during pregnancy and parturition. Histol Histopathol. 2010;25:945‐956. 20503182 10.14670/HH-25.945

[46] Romero R , Brody DT , Oyarzun E , et al. Infection and labor. III. Interleukin‐1: a signal for the onset of parturition. Am J Obstet Gynecol. 1989;160:1117‐1123. 2786341 10.1016/0002-9378(89)90172-5

[47] Hirsch E , Filipovich Y , Mahendroo M . Signaling via the type I IL‐1 and TNF receptors is necessary for bacterially induced preterm labor in a murine model. Am J Obstet Gynecol. 2006;194:1334‐1340. 16647919 10.1016/j.ajog.2005.11.004

[48] Nadeau‐Vallee M , Obari D , Quiniou C , et al. A critical role of interleukin‐1 in preterm labor. Cytokine Growth Factor Rev. 2016;28:37‐51. 26684042 10.1016/j.cytogfr.2015.11.001

[49] Chan YW , van den Berg HA , Moore JD , Quenby S , Blanks AM . Assessment of myometrial transcriptome changes associated with spontaneous human labour by high‐throughput RNA‐seq. Exp Physiol. 2014;99:510‐524. 24273302 10.1113/expphysiol.2013.072868

[50] Ritzman AM , Hughes‐Hanks JM , Blaho VA , Wax LE , Mitchell WJ , Brown CR . The chemokine receptor CXCR2 ligand KC (CXCL1) mediates neutrophil recruitment and is critical for development of experimental Lyme arthritis and carditis. Infect Immun. 2010;78:4593‐4600. 20823213 10.1128/IAI.00798-10 PMC2976349

[51] De Filippo K , Dudeck A , Hasenberg M , et al. Mast cell and macrophage chemokines CXCL1/CXCL2 control the early stage of neutrophil recruitment during tissue inflammation. Blood. 2013;121:4930‐4937. 23645836 10.1182/blood-2013-02-486217

[52] Panopoulos AD , Watowich SS . Granulocyte colony‐stimulating factor: molecular mechanisms of action during steady state and ‘emergency’ hematopoiesis. Cytokine. 2008;42:277‐288. 18400509 10.1016/j.cyto.2008.03.002 PMC2852428

[53] Gervasi MT , Chaiworapongsa T , Naccasha N , et al. Phenotypic and metabolic characteristics of maternal monocytes and granulocytes in preterm labor with intact membranes. Am J Obstet Gynecol. 2001;185:1124‐1129. 11717645 10.1067/mob.2001.117681

[54] Osman I , Young A , Ledingham MA , et al. Leukocyte density and pro‐inflammatory cytokine expression in human fetal membranes, decidua, cervix and myometrium before and during labour at term. Mol Hum Reprod. 2003;9:41‐45. 12529419 10.1093/molehr/gag001

[55] Vries MH , Wagenaar A , Verbruggen SE , et al. CXCL1 promotes arteriogenesis through enhanced monocyte recruitment into the peri‐collateral space. Angiogenesis. 2015;18:163‐171. 25490937 10.1007/s10456-014-9454-1

[56] Mobley JL , Leininger M , Madore S , Baginski TJ , Renkiewicz R . Genetic evidence of a functional monocyte dichotomy. Inflammation. 2007;30:189‐197. 17587162 10.1007/s10753-007-9036-0

[57] Martinez FO . The transcriptome of human monocyte subsets begins to emerge. J Biol. 2009;8:99. 20067595 10.1186/jbiol206 PMC2804287

[58] Hollmen M , Karaman S , Schwager S , et al. G‐CSF regulates macrophage phenotype and associates with poor overall survival in human triple‐negative breast cancer. Oncoimmunology. 2016;5:e1115177. 27141367 10.1080/2162402X.2015.1115177 PMC4839343

[59] Bizargity P , Del Rio R , Phillippe M , Teuscher C , Bonney EA . Resistance to lipopolysaccharide‐induced preterm delivery mediated by regulatory T cell function in mice. Biol Reprod. 2009;80:874‐881. 19144956 10.1095/biolreprod.108.074294 PMC2804837

[60] Olson DM , Severson EM , Verstraeten BS , Ng JW , McCreary JK , Metz GA . Allostatic load and preterm birth. Int J Mol Sci. 2015;16:29856‐29874. 26694355 10.3390/ijms161226209 PMC4691152

[61] Shynlova O , Dorogin A , Li Y , Lye S . Inhibition of infection‐mediated preterm birth by administration of broad spectrum chemokine inhibitor in mice. J Cell Mol Med. 2014;18:1816‐1829. 24894878 10.1111/jcmm.12307 PMC4196657

