# Supplementary material for: Regulation of CXCL1 chemokine and CSF3 cytokine levels in myometrial cells by the MAFF transcription factor
Source: J Cell Mol Med. 2019 Jan 22;23(4):2517–25. doi: 10.1111/jcmm.14136 (PMC6433675; doi:10.1111/jcmm.14136)
Supplement: Supplementary file 1 [file JCMM-23-2517-s001.doc]

**Table S1.** Primers for qRT-PCR and ChIP.

| Homo sapiens gene name | Forward and reverse primer sequence |
| --- | --- |
| ACTB | Fwd: 5’-TCCCTGGAAGAAGAGCTACG-3’  Rev: 5’-GTAGTTTCGTGGATGCCACA-3’ |
| PPIA | Fwd: 5’-AGACAAGGTCCCAAAGAC-3’  Rev: 5’-ACCACCCTGACACATAAA-3’ |
| TBP | Fwd: 5’-TGCACAGGAGCCAAGAGTGAA-3’  Rev: 5’-CACATCACAGCTCCCACCA-3’ |
| CSF3 (peak A) | Fwd: 5’-CTGCTCTAGTGGACACACAAATG-3’  Rev: 5’-TTTCTCCGGACTAGGCTTTG-3’ |
| CSF3 (peak B) | Fwd: 5’- ACGCTGGGGTAAATTAGGAC-3’  Rev: 5’- CGTTTTCTACAGGCCACAAG-3’ |
| CXCL1 (peak A) | Fwd: 5’- TGCAAAAATAATGCTGACACATC-3’  Rev: 5’- CCAAGCCTTCCTTCATAATGC-3’ |
| CXCL1 (peak B) | Fwd: 5’- TGCCTGTCTCAAACAGCATAC-3’  Rev: 5’- GCAAAATTTGTTTTGCTCTTTTG-3’ |
| CXCL1 (peak C) | Fwd: 5’- TGCTGAAATCTGCGTTTTTATG-3’  Rev: 5’- ATGACTTTGTCATTCCGAAAAC-3’ |
